# Spatial specificity of alpha oscillations in the human visual system

**DOI:** 10.1002/hbm.24712

**Published:** 2019-07-10

**Authors:** Tzvetan Popov, Bart Gips, Sabine Kastner, Ole Jensen

**Affiliations:** 1 Medical Faculty Mannheim, Central Institute of Mental Health, Heidelberg University, Mannheim, Germany; 2 Center for Cognitive Neuroimaging, Donders Institute for Brain, Cognition, and Behaviour, Nijmegen, The Netherlands; 3 Princeton Neuroscience Institute, Princeton University, Princeton, New Jersey; 4 Department of Psychology, Princeton University, Princeton, New Jersey; 5 Centre for Human Brain Health, School of Psychology, University of Birmingham, Birmingham, UK

**Keywords:** alpha oscillations, forward encoding modeling, magnetoencephalography, retinotopy

## Abstract

Alpha oscillations are strongly modulated by spatial attention. To what extent, the generators of cortical alpha oscillations are spatially distributed and have selectivity that can be related to retinotopic organization is a matter of continuous scientific debate. In the present report, neuromagnetic activity was quantified by means of spatial location tuning functions from 30 participants engaged in a visuospatial attention task. A cue presented briefly in one of 16 locations directing covert spatial attention resulted in a robust modulation of posterior alpha oscillations. The distribution of the alpha sources approximated the retinotopic organization of the human visual system known from hemodynamic studies. Better performance in terms of target identification was associated with a more spatially constrained alpha modulation. The present findings demonstrate that the generators of posterior alpha oscillations are retinotopically organized when modulated by spatial attention.

## 1 | INTRODUCTION

The visual system is retinotopically organized (Horton & Hoyt, 1991; Sereno et al., 1995), and this organization can even be revealed in the absence of visual stimulation when directing attention covertly to different spatial locations (Kastner, Pinsk, De Weerd, Desimone, & Ungerleider, 1999; Luck, Chelazzi, Hillyard, & Desimone, 1997; Martinez et al., 1999). Functional magnetic resonance imaging methods have provided detailed accounts of the retinotopic maps underlying this topographic organization (Wang, Mruczek, Arcaro, & Kastner, 2015). While the retinotopy in humans is typically derived from fMRI, its relationship to animal or human electrophysiology is a matter of continuous scientific debate (Singh, 2012). Several studies highlight a relationship between the hemodynamic response and oscillatory activity (Hermes, Nguyen, & Winawer, 2017; Logothetis, Pauls, Augath, Trinath, & Oeltermann, 2001; Scheeringa et al., 2011; Scheeringa, Koopmans, van Mourik, Jensen, & Norris, 2016). It is a common finding that deployment of attention to either the left or right hemifield results in a decrease contralateral to the attended hemifield and a relative ipsilateral increase of alpha band power (Foxe, Simpson, & Ahlfors, 1998; Kelly, Lalor, Reilly, & Foxe, 2006; Sauseng et al., 2005; van Dijk, van der Werf, Mazaheri, Medendorp, & Jensen, 2010; Worden, Foxe, Wang, & Simpson, 2000). While this hemispheric modulation is well established, it is not clear whether the distribution of the modulated alpha generators shows further retinotopic organization. That is, alpha power associated with the allocation of attention toward a particular visual polar angle may be retinotopically distributed such that the focus of attention is indicated by a local depression in power of the associated alpha generators. To date, few studies have addressed the variation of alpha amplitude as a function of visual polar angle (Bahramisharif, van Gerven, Heskes, & Jensen, 2010; Rihs, Michel, & Thut, 2007). While these studies have demonstrated that the direction of attention can be predicted based on the topographic distribution in the alpha band, the distribution of the neocortical generators was not estimated.

In a recent study, we used a modified version of the Eriksen– Flanker paradigm to elucidate the neurophysiological spatiotemporal activity associated with visuospatial attention using MEG in humans (Popov, Kastner, & Jensen, 2017). In a nonhuman primate study, it has been shown that modulations of alpha band dynamics were associated with the deployment of visuospatial attention (Saalmann, Pinsk, Wang, Li, & Kastner, 2012). Participants covertly attended a location indicated by a cue at which a subsequent target appeared in a circular array. During the delay period, attentional allocation has been shown to modulate alpha-band activity in both human and nonhuman primates (Popov et al., 2017; Saalmann et al., 2012). In the present report, we asked to what extent the modulations of alpha-band activity were distributed retinotopically. In order to do so, we used the techniques of *spatial location tuning functions* (Anderson, Serences, Vogel, & Awh, 2014; Brouwer & Heeger, 2009; Ester, Anderson, Serences, & Awh, 2013; Ester, Sprague, & Serences, 2015; Freeman, Brouwer, Heeger, & Merriam, 2011; Garcia, Srinivasan, & Serences, 2013; Kay, Naselaris, Prenger, & Gallant, 2008; Serences & Saproo, 2012; Serences, Saproo, Scolari, Ho, & Muftuler, 2009; Sprague, Saproo, & Serences, 2015; Sprague & Serences, 2013) in combination with a beamforming approach.

## 2 | MATERIALS AND METHODS

### 2.1 | Participants

Thirty participants (15 female, mean age 27.7 ± 8.8 years) without a history of neurological and/or psychiatric disorders signed a written informed consent form prior to the experiment in accordance with the Declaration of Helsinki. The study was approved by the local ethics committee (commission for human-related research CMO-2014/288 region Arnhem/Nijmegen NL). This data set has been used for a previous report (Popov et al., 2017) that focused on different aspects of analyses.

### 2.2 | Stimulus material and procedure

**FIGURE 1:**
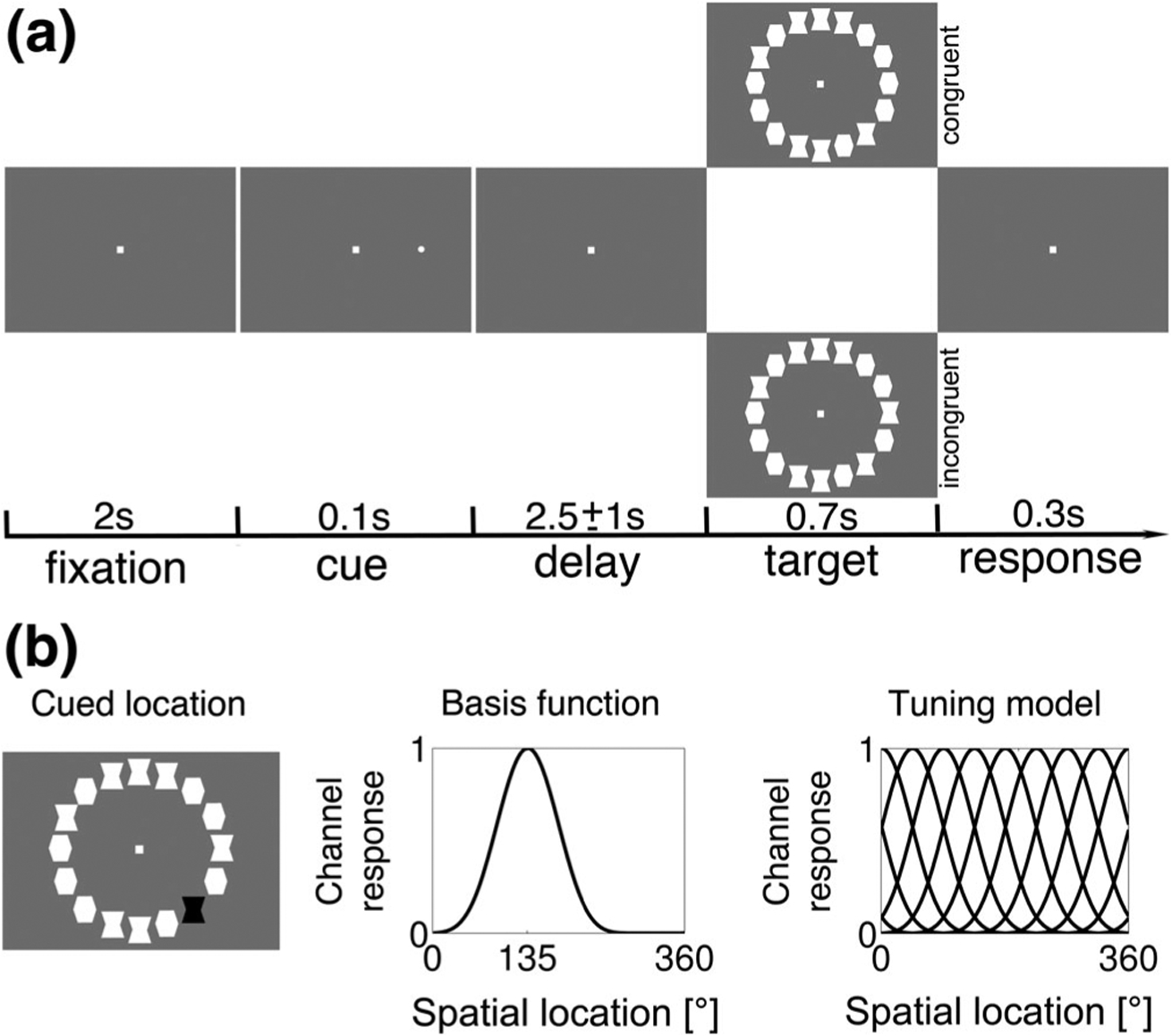
(a) After a fixation interval (2 s), a visual cue was presented (0.1 s) at one of 16 locations, indicating the presentation location of an upcoming target that had to be maintained during a delay interval of 0.1–3.5 s. Upon presentation of a circular stimulus array, participants indicated with a button press whether the target at the cued location was a bowtie or a barrel-shaped stimulus. Target stimuli were flanked by either congruent or incongruent neighboring stimuli. (b) Central assumption of the forward encoding model. Maintenance of a cued position (e.g., 135° left) is associated with an idealized tuning curve (basis function), modeled as a half-wave rectified and squared sinusoid (middle). The brain activity at a given MEG sensor/voxel was fitted with a tuning model (right) representing the weighted sum of eight basis functions (16 locations binned into two) evenly spaced around the 0–360° visual field space

A variant of an Eriksen flanker task was used (Saalmann et al., 2012). In each trial, participants were instructed to fixate on a central white square (0.82° visual angle) presented on a gray background at a distance of approximately 84 cm. After a baseline “fixation” period (2 s, Figure 1a), a spatial cue (100 ms duration; 1.36° visual angle) was presented at one of 16 pseudorandomly chosen locations (Figure 1a). After a delay interval of 2.5 ± 1 s, during which subjects maintained the location information, a target shape (barrel or bowtie) appeared at the precued location embedded in a circular array of 16 shape stimuli (radius 16.5 cm, visual angle 22.22°). Targets were flanked either by congruent (C) or incongruent (IC) distracters. Targets and distracters had a visual angle of 2.73° × 3.07° . Target type (barrel or bowtie, 50% chance) as well as distractor congruency (congruent and incongruent at 50% chance) were randomly determined on each trial. Participants indicated via button press whether the target was a “barrel” (left index finger) or a “bowtie” (right index finger).

Visual stimuli were back-projected onto a semitranslucent screen by an Eiki LC-XL100L projector with a projection resolution of 1,024 × 768 pixels. Eye movements and blinks were continuously recorded using an Eyelink 1000 eye-tracking device (SR Research, Ontario, Canada).

### 2.3 | Data acquisition

Neural activity was monitored by a whole-head 275-sensor axial-gradiometer system (Omega 2000, VSM MedTech Ltd., Port Coquitlam, BC, Canada). The data was sampled at 1.2 KHz after a 400 Hz low pass filter was applied. Anatomical magnetic resonance imaging (MRI) images were acquired using a 1.5 T Siemens Magnetom Avanto system (Erlangen, Germany) following the MEG session.

### 2.4 | Behavioral data analysis

The flanker effect was quantified in terms of accuracy and reaction time (RT). The stronger distractibility by incongruent than congruent flankers was assessed as the difference in RT between the congruent and the incongruent conditions. This effect was further quantified in terms of effect size (Cohen’s *d*) and related to neuronal data using Spearman’s rank order correlations.

### 2.5 | Neural data analysis

All offline aspects of data analysis concerned with spectral decomposition, source analysis and quantification of alpha lateralization are identical to (Popov et al., 2017). On average 553 ± 86 out of 640 (40 per location) trials entered the analysis. Based on visual inspection, epochs contaminated by strong muscle movement, and/or sensors “jump” artifacts were rejected from further analysis. Thereafter, an independent component analysis (ICA; Jung et al., 2001) was applied in order to remove components associated with eye blink and cardiac activity.

#### 2.5.1 | Spectral analysis

Spectral analysis was computed for each trial using a Fast Fourier Transformation (FFT) approach. A sliding Hann window (500 ms long) was used. The time window advanced in 50 ms increments and the spectral resolution was 2 Hz.

Spectral power was calculated for the horizontal and vertical components of the estimated planar gradient for each sensor location and subsequently summed (Bastiaansen & Knosche, 2000). This step usually simplifies the interpretation of the signal topography, since maxima typically are observed above a given source (Hämäläinen, Hari, Ilmoniemi, Knuutila, & Lounasmaa, 1993).

#### 2.5.2 | Source analysis

The time-series were identified at source level by applying a time- domain spatial filtering algorithm (LCMV, linearly constrained minimum variance; Van Veen, van Drongelen, Yuchtman, & Suzuki, 1997). This algorithm uses the covariance matrix of the MEG data to construct a spatial filter for a given location (grid point). These spatial filters were estimated on the basis of all trials and for the [−1,0] s interval just prior to stimulus onset. Subsequently, these filters were applied to the data in order to estimate the time series for a given location. After identification of the fiducials, the nasion, left and right preauricular points, coregistration with Montreal Neurological Institute (MNI) coordinates were applied. A realistic, single-shell brain model (Nolte, 2003) was constructed based on the anatomical MRI. Forward solution for each participant was estimated using a common dipole grid (10 mm^3^ grid) in MNI space, warped onto each participant’s anatomy.

#### 2.5.3 | Forward encoding model

A forward encoding model approach was used following the procedures, as initially described in Brouwer and Heeger (2009, 2011). The general assumption is that a given MEG sensor (denoted as *m*) records distributed activity generated within the brain volume (denoted here as A). In order to find the activity patterns related to spatial attention, we attempt to decode the direction of attention from the MEG signals. In the present report, the 16 locations (Figure 1a) were combined in groups of two resulting in eight angles equally spaced within the 360° visual field. Based on these eight angles, we constructed eight corresponding basis functions (Figure 1b, right). Each of these basis functions is constructed to resemble an idealized tuning curve (Figure 1b, middle). For each stimulus location (*l*), MEG sensor (*m*), and repetition (*n*) alpha power values (8–13 Hz) during the delay period (−1–0 s thus prior to target array onset at 0 s) were extracted and sorted into six equal subsets each of which sampled equally from each location to perform a sixfold cross-validation. In the cross-validation, we used 5/6 of the data as a training subset $B_{1}\left(m\times n_{\text{training}}\right)$ and 1/6 of the data as a test subset $B_{2}\;\left(m\times n_{\text{test}}\right)$. A set of weights W(m×l) that allowed us to decode the response matrix ${\mathbb{C}}_{1}\;\;\left(l\times n_{\text{training}}\right)$ from the training subset $B_{1}$ was determined through the use of a linear model of the form:

(1) $${\mathbb{C}}_{1}\;=\;WB_{1}\;+\;\epsilon .$$

Where *ϵ* contains (assumed Gaussian) error terms that we wish to minimize. To this end, we used ordinary least-squares regression to estimate the weight matrix W(m×l):

(2) $$W\;=\;{\mathbb{C}}_{1}^{T}B_{1}{\left(B_{1}B_{1}^{T}\right)}^{-1}.$$

Based on this weight matrix and on the test data $B_{2}$ an estimated response matrix ${\hat{\mathbb{C}}}_{2}\left(l\times n_{\text{test}}\right)$ was calculated:

(3) $${\hat{\mathbb{C}}}_{2}\;=\;WB_{2}.$$

**FIGURE 2:**
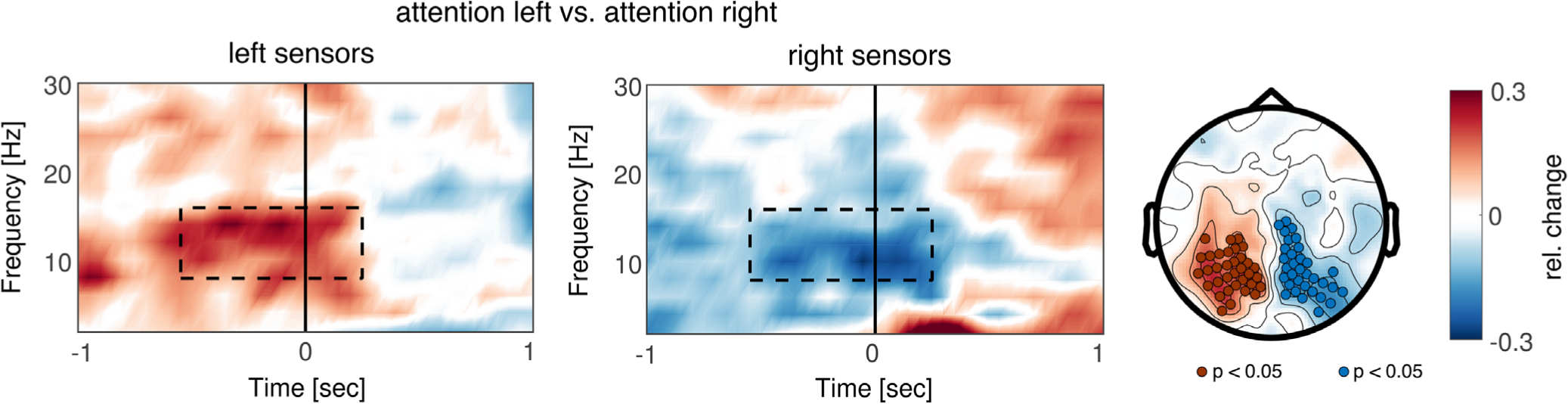
Time-frequency representation of power reflecting the contrast between left and right cued trials ((power_left_attention_ – power_right_attention_)/(power_left_attention_ + power_right_attention_)). Alpha power decreased in sensors contralateral to the direction of attention (right sensors) and increased in ipsilateral sensors (left sensors). The topography (right) illustrates the 8–13 Hz alpha power modulation during the delay interval (−1–0 s, dashed box). The marked sensors indicate clusters of sensors revealing significant differences after controlling for multiple comparisons

Decoded channel responses ${\hat{\mathbb{C}}}_{2}$ were circularly shifted such that estimates associated with locations that evoked a response were positioned at 0° of the location space spanning −180° to 180°. This additional step facilitates the interpretation of the results since all estimated responses are aligned to a common center. Accurate model is characterized by a maximum at 0° and a minimum at −180° = 180°, whereas an inaccurate model fit approximates a flat line (Figure 2b). This procedure was repeated until each subset had served as training and test set.

To interpret our weights W, we transformed them into activation patterns A of a corresponding forward encoding model (Haufe et al., 2014):

(4) $$A\;=\;\Sigma_{B_{1}}\;W^{T}\Sigma_{{\hat{\text{C}}}_{1}}$$

where $\Sigma_{B_{1}}\;=\;\text{cov}\left(B_{1}\right)$ and $\Sigma_{{\hat{C}}_{1}}\;=\;\text{cov}\left({\hat{C}}_{1}\right)\;=\;\text{cov}\left(WB_{1}\right)$ are covariance matrices. The advantage of using A over the raw weights W is that elements of W may reflect suppression of “signals of no interest” (Haufe et al., 2014). That is, W may reflect correlations across sensors in $B_{1}$ caused by noise and therefore do not reflect brain activity related to the signals we want to decode $\left({\mathbb{C}}_{1}\right)$. Transforming to activation patterns A mitigates this problem.

Forward encoding modeling was performed on both sensor and source space. The source space analysis was performed in order to map the activation patterns onto the individual brain volume. Subsequently, all source topographies were averaged and thresholded using the probabilistic atlas of visual topography in human cortex (Wang et al., 2015).

### 2.6 | Statistical analysis

Statistical quantification of the neuronal data was carried out by a cluster-based approach with Monte Carlo randomization (Maris & Oostenveld, 2007). The Monte Carlo estimate identifies clusters of activity assuming that the null hypothesis (i.e., that the data is exchangeable) can be rejected while controlling for multiple comparisons. Relationships between neuronal data and behavioral performance were explored through Spearman’s rank correlation coefficients.

## 3 | RESULTS

### 3.1 | Amplitude modulation of alpha oscillations during the delay indexes the direction of attention to a hemifield

Time–frequency analyses of power confirmed effects in the alpha frequency band during the delay interval (Figure 2). Maintenance of a cued position in the left visual hemifield was associated with a sustained contralateral posterior alpha band power decrease (Figure 2, right, *p* < .05 cluster-based permutation test) accompanied by ipsilateral power increase (*p* < .05, Figure 2, middle) over posterior occipital sensors. Previous analyses of this effect demonstrated that the sources were confined to early visual brain areas (Popov et al., 2017). These results confirm the well-known hemispheric lateralization of alpha-band activity and were taken as a starting point to investigate the sources of alpha-band activity associated with the 16 different directions.

### 3.2 | Amplitude modulation of alpha activity allows for decoding of spatially selective tuning functions

**FIGURE 3:**
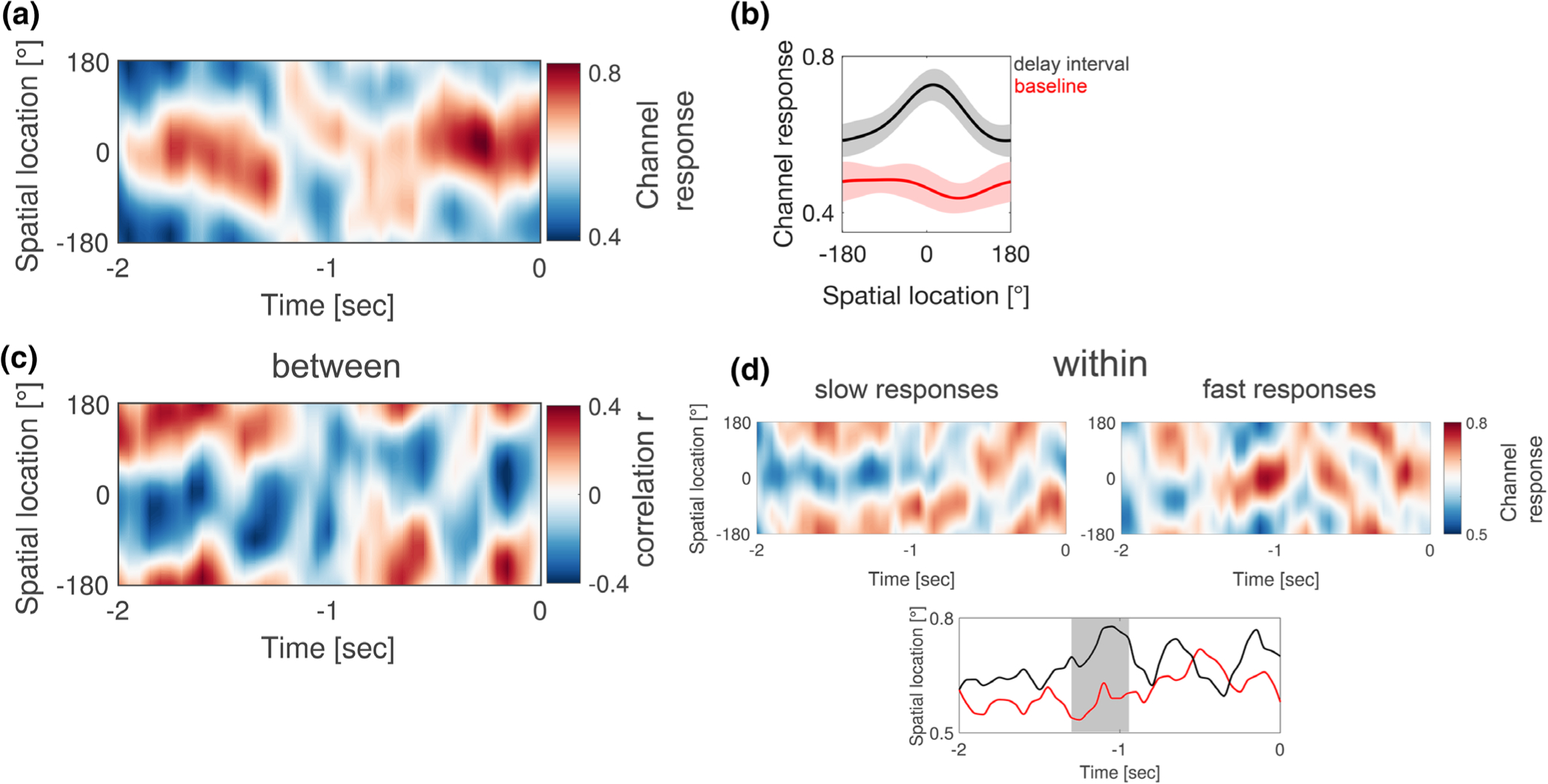
Visual field selectivity of alpha power fluctuations. (a) Location tuning functions exhibit a clear peak at 0° (ordinate) during the delay interval −2 to 0 s (abscissa). (b) Location tuning responses averaged across time for the delay interval (black) and a baseline interval of 1 s prior to cue onset (red). There was a significant condition difference (*p* < .05, cluster-based permutation test). Shaded areas illustrate SEM. (c) Time-resolved correlation averaged across subjects between channel tuning responses and effect size (Cohen’s *d*) on the RT difference between incongruent and congruent trials. Stronger visual field selectivity during the delay interval was associated with lower behavioral cost induced by the incongruent neighboring stimuli. (d) Time-resolved location tuning functions within individual slow (left) and fast (right) responses. Left graph represents the grand mean of the decoding across subject’s slow trials and right graph the respective grand mean for subject’s fast trials. Bottom graph summarizes the time courses of the channel responses averaged around 0°. Shaded area highlights the temporal cluster of the basis of which H0 was rejected

The location selectivity of alpha oscillations was identified by a linear forward encoding model that estimated the magnitude of the response in each MEG sensor as a weighted sum of eight idealized location tuning functions (each two locations are grouped, see section 2). The estimates of the relative magnitude of alpha oscillations tuned to different locations in the visual field space are illustrated in Figure 3a. The responses to the different locations were aligned such that each location response was shifted to zero degrees in order to allow for condition averaging and statistical evaluation. The channel tuning responses were enhanced selectively responding to the particular visual field location paralleled by a bell-shaped drop-off reflecting direction tuned neural populations. This visual field selectivity was specific to the delay interval as it was absent in the precue baseline interval (−4 to −3 s, Figure 3b). Importantly, the magnitude of these spatial location tuning functions was behaviorally relevant addressed in the following section.

### 3.3 | Magnitude of spatial location tuning functions is related to distracter filtering

Incongruent flankers are associated with a reaction time increase and accuracy decrease (Popov et al., 2017). This effect can be quantified as the RT difference between incongruent and congruent conditions resulting in one behavioral measure per subject. The 30 measures can then be related to their corresponding tuning functions (time × location representations, Figure 3a) resulting in a time × location representation of the correlation between tuning functions and the behavioral manifestation of the Flanker effect. This is illustrated in Figure 3c. Participants, who were less distracted by the incongruent flankers were better in maintaining the regional specific modulation of the alpha activity as confirmed by the tuning curves (*p* < .05, cluster-based permutation test, Figure 3c). In addition, this behavioral relevance can be substantiated on a within-trial basis. For a given participant, the individual trials per location were grouped into slow and fast responses (median split). Subsequently, tuning functions can be re-computed for the slower and faster responses respectively. Fostering signal-to-noise ratio this analysis has been performed in source space. Figure 3d summarizes the result of this analysis highlighting that in addition to magnitude, the sustainability of the location tuning response predicts the speed of upcoming responses. Prolonged tuning to the location at which the target is about to occur precedes faster responses (*p* < .05, cluster-based permutation test, Figure 3d, right). In contrast, slower responses are characterized by “looser” location tuning that is re-instantiated prior to the onset of the target (Figure 3d, left).

### 3.4 | Alpha amplitude tuning functions specific to visual field location reveal relation to retinotopic maps

**FIGURE 4:**
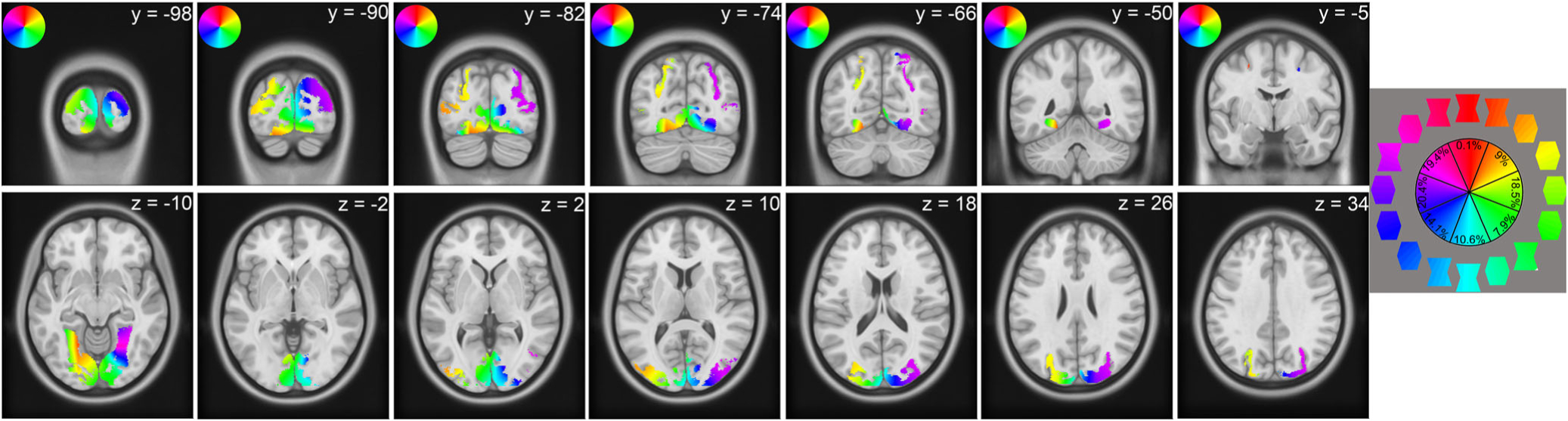
Group averaged phase maps of coronal (top) and transversal (bottom) slides. The color code denotes the corresponding visual field angle (and thus location; see lower right plot). The proportion of grid points coding for a given angle is shown at right. Activation patterns were thresholded using the probabilistic atlas of visual topography in human cortex (Wang et al., 2015) parcels overlapping with the atlas are opaque, nonoverlapping transparent

For each grid point, activation patterns A were mapped onto the cortical volume. Isopolar angle maps were created in accordance with the probabilistic human visual system atlas (Wang et al., 2015) regions overlapping with atlas parcels are opaque and nonoverlapping are transparent. The results of this analysis are illustrated in Figure 4. The color scale indicates stimulus location according to the polar angle of the visual field representation. In agreement with previous mapping studies in humans (Sereno et al., 1995; Wang et al., 2015), the retinotopic maps alternate between vertical and horizontal meridians in both ventral and dorsal directions in early visual cortex and between upper and lower vertical meridian representations in higher-order cortex. The upper visual field vertical meridian is color-coded in magenta–red–orange, the horizontal corresponds to purple–light green and the lower field vertical meridian is indicated by blue–light blue–green color.

## 4 | DISCUSSION

In the present report, participants performed a variant of the Eriksen– Flanker task in which spatial attention was allocated in 16 directions. Alpha-band activity during the delay period was tuned to the specific visual field location. The magnitude of the tuning was related to less distractibility by interfering input indicating not only the spatial specificity of alpha-band activity but also its relevance to behavior.

Evidence from fMRI and electrophysiology studies in humans and nonhuman primates indicate that the borders of vertical meridian representations span between ventral and dorsal V1–V2 and dorsal V3–V4 borders while horizontal meridian representations expand around the fundus of the calcarine sulcus, ventral V2, and dorsal V2–V3 borders (Felleman & Van Essen, 1991; Newsome, Maunsell, & Van Essen, 1986; Sereno et al., 1995; Silver & Kastner, 2009; Tootell, Hamilton, & Silverman, 1985; Tootell, Silverman, & De Valois, 1981; Van Essen, Newsome, Maunsell, & Bixby, 1986; Wandell & Winawer, 2011). While this literature reports topographic organization following visual stimulation, reports on retinotopic organization of spatial attention in the absence of visual stimulation are less frequent (Kastner et al., 1999). Topographic maps of spatial attention are typically reported for parietal regions such as the intra-parietal sulcus IPS1 and IPS2 (Saygin & Sereno, 2008; Silver & Kastner, 2009; Silver, Ress, & Heeger, 2005; Szczepanski, Konen, & Kastner, 2010). The present analysis demonstrates that this functional organization is reflected in the modulation of ongoing oscillatory brain activity, reflecting a topographic organization including early visual and parietal. During the delay period, in the absence of an external stimulus, the spatial distribution of alpha activity reflected the participant’s attended location in the visual field. In line with previous electrophysio-logical studies (Bahramisharif et al., 2010; Cicmil, Bridge, Parker, Woolrich, & Krug, 2014; Fahrenfort, Grubert, Olivers, & Eimer, 2017; Hagler Jr. et al., 2009; Kelly et al., 2006; Kelly, Gomez-Ramirez, & Foxe, 2009; Perry et al., 2011; Rihs et al., 2007), the present report points to a novel mechanism on the relevance of alpha activity in allocating neurocomputational resources in a spatial attention task. This mechanism relates to the notion that the spatial variability of alpha power reflects a “moving spot” of reduced alpha (Jensen, Gips, Bergmann, & Bonnefond, 2014; Jensen & Mazaheri, 2010). The reduced alpha power might then reflect a gain increase of the attended visual field. This insight challenges a view in which alpha power modulations are rather coarse and potentially reflects the engagement and disengagement of an entire hemi-sphere or at best several brain areas at once. Our present results suggest that alpha power modulations can be focal and relate to underlying retinotopic organization. These modulations are due to internal attention mechanisms rather than external stimulus input. This is in line with recent findings from human and animal intracranial recordings demonstrating the spatial specificity of alpha activity in the auditory domain. Task-relevant alpha power is differentially modulated within the auditory system in accordance with differential sound input and relates to population-level activity (de Pesters et al., 2016). Thus, the spatial tuning reported here potentially reflects the allocation of spatial attention by setting the gain in a regionally specific sense such that processing of upcoming targets are facilitated while distractors are inhibited. Indeed, the ability to modulate the alpha activity to maintain attention at a particular location had behavioral consequences: the greater the OTF, the smaller was the increase of RT in the incongruent condition, thus resulting in a decrease of the behavioral cost in the flanker paradigm (Figure 3c). It is conceivable that local alpha modulation has direct consequences for the allocation of computational resources where the phase of the alpha activity is reported to modulate firing rate locally (Haegens, Nacher, Luna, Romo, & Jensen, 2011). In addition, amplitude reduction is hypothesized to enlarge the duty cycle for processing of incoming input (Jensen et al., 2014). Accordingly, during the delay interval, location-specific tuning of alpha oscillations ensure engagement of task-relevant brain areas while inhibiting areas processing the unattended locations (Jensen & Mazaheri, 2010; Figure 2). In sum, we suggest that the neural mechanisms of spatial attention include a focal topographically organized depression of alpha power effectively controlling neural gain function and distractor interference.

Previous MEG studies have reported retinotopic organization of primary visual areas following spatially-specific visual input (Cicmil et al., 2014; Hagler Jr. et al., 2009; Perry et al., 2011). Here we extend this literature by demonstrating retinotopic specificity beyond early visual areas in an absence of visual input, extending to parietal regions. Parietal regions and the frontal eye fields have been related to a top-down modulatory control within a variety of cognitive tasks and imaging modalities [for review, please refer Paneri and Gregoriou (2017), Shomstein (2012), and Silver and Kastner (2009)]. In line with the fMRI literature, ventral, dorsal, and parietal cortices displayed specialized location tuning properties (Figure 4). We suggest that decreases of alpha activity that are specific to spatial attention potentially lengthen the oscillatory duty cycle, thereby preparing the corresponding task-relevant neural circuit for faster and accurate performance of an upcoming target. In contrast, frontal eye fields were tuned, at least to some extent, to visual field locations along the horizontal meridian (Figure 4) reflecting a rather sparse representation “overseeing” the principal direction of attention potentially regulating activity in early visual areas in a top-down fashion (Bressler, Tang, Sylvester, Shulman, & Corbetta, 2008; Ekstrom, Roelfsema, Arsenault, Bonmassar, & Vanduffel, 2008; Ekstrom, Roelfsema, Arsenault, Kolster, & Vanduffel, 2009; Marshall, O’Shea, Jensen, & Bergmann, 2015; Moore & Armstrong, 2003; Popov et al., 2017). However, it should be noted that the spatial resolution of the MEG at that area is lower as compared to visual areas such that this interpretation should be taken with caution. Future research, utilizing individual head casts reducing head motion and thus increasing sensitivity would be of great value in addressing this issue. Finally, to what extent, alpha-oscillatory activity in early visual areas is capable of tracking retinal eccentricity and thereby subsequent target processing should be addressed in future studies. A substantial body of literature suggests that perception depends on amplitude and phase properties of ongoing oscillatory activity in the alpha bands. An affirmative case of eccentricity tuning reflected in alpha activity might bring us a step closer to a mechanistic account of visual perception and attention.
